# Supplementary material for: The MitoLuc Assay System for Accurate Real-Time Monitoring of Mitochondrial Protein Import Within Mammalian Cells
Source: J Mol Biol. Author manuscript; Available in PMC 2024 Aug 23. (PMC7616392; doi:10.1016/j.jmb.2023.168129)
Supplement: Supplementary material [file EMS198153-supplement-Supplementary_material.docx]

The MitoLuc Assay System for Accurate Real-Time Monitoring of Mitochondrial Protein Import within Mammalian Cells

Hope I Needs, James S Lorriman, Gonçalo C Pereira^*^, Jeremy M Henley and Ian Collinson^†^

*School of Biochemistry, University of Bristol, Bristol BS8 1TD, UK*

^†^, corresponding author: [ian.collinson@bristol.ac.uk](mailto:ian.collinson@bristol.ac.uk)

^*^, Nanna Therapeutics, Merrifield Centre, Rosemary Lane, Cambridge, CB1 3LQ, UK

**Supplementary Figures S1 – S4**

**Fig. S1.** Average traces with standard deviation from the MitoLuc import assay showing the biological replicability of import in the presence of various dilutions of furimazine (Fz; dilutions indicated as ratios of furimazine: assay buffer). Fz was applied to cells for 5 min prior to starting the assay, the MitoLuc assay was then carried out with the Su9-EGFP-pep86 precursor protein. Background was removed and data was normalised to cellular eqFP670 expression, and the maximum amplitude from the run, to allow comparison between runs regardless of raw values. N=4 biological replicates, each with n=3 technical replicates.

**
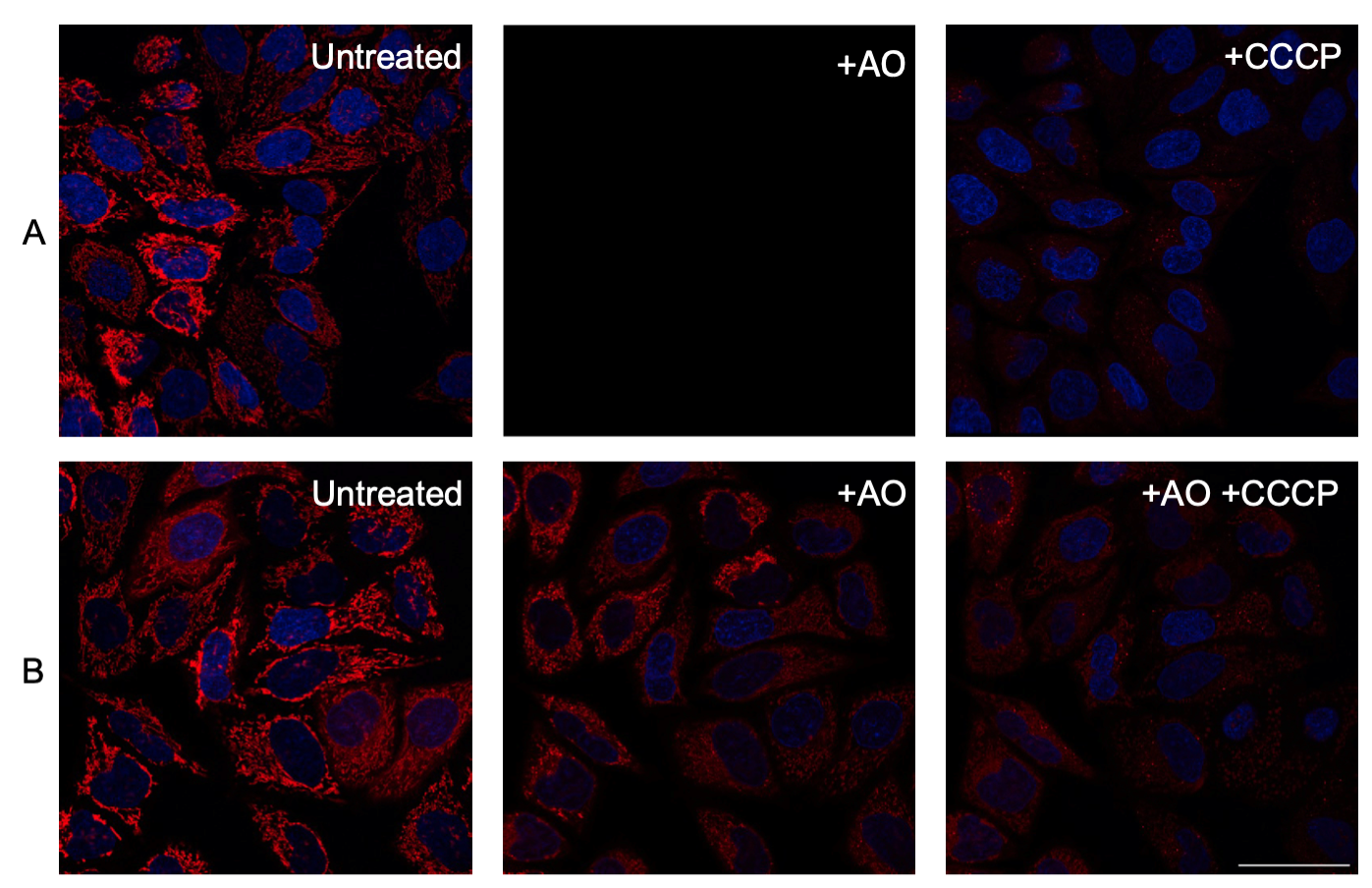
**

Fig. S2. TMRM fluorescence demonstrating the membrane potential of HeLa cells in the absence or presence of AO and CCCP. Membrane potential is shown by TMRM staining (red), and nuclei are stained with Hoechst 33342 (blue). (A) Cells were imaged by live cell confocal microscopy in the absence of inhibitors for 10 minutes (untreated, top left panel), followed by the addition of 10 µM CCCP, and imaging of the same cells for a further 10 minutes (+CCCP, top right panel). (B) Cells were imaged by live cell confocal microscopy in the absence of inhibitors for 10 minutes (untreated, bottom left panel), followed by the addition of 1 µM antimycin A and 5 µM oligomycin, and a further 10 minutes of imaging the same cells (+AO, bottom middle panel). Then, 10 µM CCCP was added, and the same cells were imaged for a further 10 minutes (+AO +CCCP, bottom right panel). Representative images are shown. Error bar is 50 µm. N=3 biological replicates.

**

**Fig. S3.** MitoLuc import trace for ACP1-pep86. HeLa cells expressing eqFP670-P2A-Cox8a-11S were incubated in MitoLuc assay buffer for 5 minutes prior to monitoring the import of the ACP1-pep86 precursor using the MitoLuc assay. Background was removed and data was normalised to cellular eqFP670 expression, and the maximum amplitude from the run, to allow comparison between runs regardless of raw values. The resulting normalised average trace with error bars representing SD are shown. N=3 biological replicates, each with n=3 technical replicates.

**Fig. S4.** Western blotting-based import assay to analyse the accumulation of Su9-EGFP-pep86 in the mitochondria of HeLa cells. HeLa cells were subjected to rPFO permeabilization followed by incubation with Su9-EGFP-pep86 in MitoLuc assay buffer. Import was quenched by the addition of VOA (valinomycin, antimycin A, and oligomycin) at 0, 5, 10, 15, 20, and 30 min and mitochondria were isolated and analysed by Western blotting for the accumulation of full-length Su9-EGFP-pep86 and the presequence cleaved mature version (EGFP-pep86). The ratio of mature to full-length precursor was used as a measure of import. **(A)** shows a representative Western blot against GFP (Chromotek 3H9). **(B)** shows the average import trace for Su9-EGFP-pep86 by Western blotting (N=3 biological replicates, error bars represent SD) and **(C)** shows a representative import trace for Su9-EGFP-pep86 by MitoLuc assays, for comparison.
