# Supplementary material for: The MitoLuc Assay System for Accurate Real-Time Monitoring of Mitochondrial Protein Import Within Mammalian Cells
Source: J Mol Biol. Author manuscript; Available in PMC 2024 Aug 23. (PMC7616392; doi:10.1016/j.jmb.2023.168129)
Supplement: Appendix [file EMS198153-supplement-Appendix.pdf]

Appendix A. Supplementary Data

Supplementary data to this article can be found online at https://doi.org/10.1016/j.jmb.2023.168129.
